# Mechanochemistry

**DOI:** 10.3762/bjoc.13.234

**Published:** 2017-11-07

**Authors:** José G Hernández

**Affiliations:** 1 Institute of Organic Chemistry, RWTH Aachen University, Landoltweg 1, D-52074 Aachen, Germany

**Keywords:** green chemistry, mechanochemistry, organic chemistry, solvent-free

The scientific community’s general interest in using mechanical energy to trigger or facilitate chemical reactivity has been growing at a speedy pace. In particular, in recent years, organic chemistry has witnessed a constant flow of examples where the use of mechanochemical techniques proved not only to outperform traditional solution-based methodologies, but also enabled access to otherwise impossible chemical reactivity in many cases. From a green chemistry perspective, mechanochemical activation conducted by milling, shearing, pulling or ultrasonic irradiation allows for the possibility to drastically reduce the amount of solvent needed during chemical reactions, even to the point of achieving chemical reactivity under solvent-free conditions. Additionally, the utilization of mechanochemical technology can often further simplify the posterior work-up procedures, having a deeper impact on the sustainability of the global synthetic process (reduction of waste, lower energy consumption, absence of external heating, fast reactivity, etc.).

When compared with other, more established alternatives to carry out chemical transformations, mechanochemistry can still be considered as a nascent approach. Therefore, Thematic Series like this one gathers works from experts on the topic to encourage the chemistry community to adopt the concepts of mechanochemistry, and secondly, it strengthens the field. In addition to the previous special issues dedicated to mechanochemistry published in other peer-reviewed scientific journals [1–2], the *Beilstein Journal of Organic Chemistry* sought to host a Thematic Series specifically covering the field of organic mechanochemistry. Altogether, this Thematic Series contains more than two dozen papers from colleagues working in at least fifteen different countries across America, Asia, Africa and Europe. As a consequence, the fantastic job by the *Beilstein Journal of Organic Chemistry* editorial and production teams, authors and reviewers will definitely help in the consolidation of mechanochemistry worldwide.

While exploring this Thematic Series, the reader will find a substantial collection of papers where the advantages of mechanosynthesis are demonstrated throughout the numerous full research papers and highlighted in the review articles that complement this Thematic Series. These contributions are set to become the background knowledge for future applications in the field, which are anticipated to continue to push the boundaries of mechanochemistry further and beyond.

José G. Hernández

Aachen, September 2017
